# HIVToolbox, an Integrated Web Application for Investigating HIV

**DOI:** 10.1371/journal.pone.0020122

**Published:** 2011-05-25

**Authors:** David Sargeant, Sandeep Deverasetty, Yang Luo, Angel Villahoz Baleta, Stephanie Zobrist, Viraj Rathnayake, Jacqueline C. Russo, Jay Vyas, Mark A. Muesing, Martin R. Schiller

**Affiliations:** 1 School of Life Sciences, University of Nevada Las Vegas, Las Vegas, Nevada, United States of America; 2 Aaron Diamond AIDS Research Center, New York, New York, United States of America; 3 Department of Molecular, Microbial, and Structural Biology, University of Connecticut Health Center, Farmington, Connecticut, United States of America; Virginia Tech, United States of America

## Abstract

Many bioinformatic databases and applications focus on a limited domain of knowledge federating links to information in other databases. This segregated data structure likely limits our ability to investigate and understand complex biological systems. To facilitate research, therefore, we have built HIVToolbox, which integrates much of the knowledge about HIV proteins and allows virologists and structural biologists to access sequence, structure, and functional relationships in an intuitive web application. HIV-1 integrase protein was used as a case study to show the utility of this application. We show how data integration facilitates identification of new questions and hypotheses much more rapid and convenient than current approaches using isolated repositories. Several new hypotheses for integrase were created as an example, and we experimentally confirmed a predicted CK2 phosphorylation site. Weblink: [http://hivtoolbox.bio-toolkit.com]

## Introduction

Human immunodeficiency virus type 1 (HIV-1) is a recently emerged human virus that over the past thirty years has ignited the worldwide AIDS pandemic [1]. Extensive characterization of the viral enzymatic machinery has led to a collection of drugs that inhibit each of the respective activities of these proteins and when used in combination have curtailed overt viral replication in infected individuals [2]. Despite these advancements, patients undergoing these therapeutic regimens can develop drug resistant viral strains leading to higher viral loads and further disease progression. Identification of new viral and/or host drug targets is warranted to place further barriers to new cycles of viral replication.

To understand the complex processes involved during viral infection, we assert that it is beneficial to consider all available knowledge to effectively select targets for therapeutic intervention. These include molecular information about protein sequence and structure, protein-protein interactions, protein modifications, protein localization, protein domains and phylogenetic information. Several databases have emerged which focus on subsets of the aforementioned areas and are routinely used by scientists to study HIV [3]–[7].

These and other bioinformatic databases and applications generally focus on a specific area of knowledge, and are federated with some information from other databases. These segregated data sources likely limit the ability to investigate and understand complex biological systems. Here, we compile existing informatics relating to HIV-1 infection into an intuitively accessed database. We propose that integrated data management has distinct advantages over existing data repositories in hypothesis-generated science and experimental interpretation. We have built HIVToolbox, a database/web application that integrates information about HIV protein sequence, structure, and function. This tool facilitates hypothesis generation, experimental design, and interpretation as demonstrated by example analyses of HIV integrase.

## Results

### Examples of analyses with HIVToolbox

**Figure 1:**
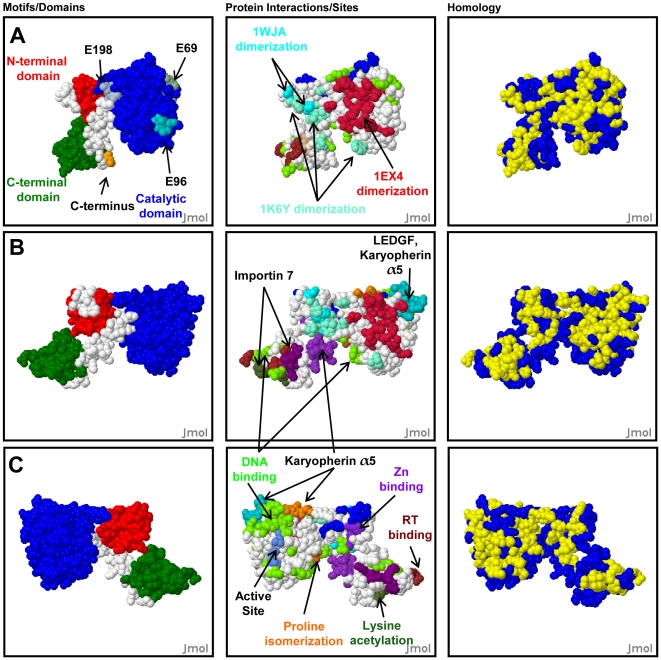
Analysis of Integrase with HIVToolbox. (**A–C**) Output of HIVToolbox showing relationships of IN elements shown in a model constructed from superposition of the catalytic domains in structures 1EX4 and 1K6Y. Residues 1–7, 47–55, 140–148, and 270–288 are unstructured and not shown. The left panels shows domains [NTD (red), CCD (Blue), and CTD (green)] and minimotifs; the center panels show functional sites and protein-protein interactions the right panels shows residues that are >98% conserved in 3787 HIV-1 IN isolates (yellow). (**A**) Location of three of the four putative CK2 phosphorylation sites located on the surface of the IN CCD (left panel); the 4^th^ CK2 site is in the CTD unstructured region. Numbers indicate the positions of putative phosphorylation sites. D270 is the last residue in the structure (orange). Conservation of the residues on CK2 sites is shown in **Table 1**. (**B**) Conservation and location of the dimerization interface(s). Residues at the dimerization interface less than 3.25 Å from atoms in the other chain are colored: (red, 1EX4), (cyan, 1WJA), and (lighter cyan, 1K6Y). (**B, C**) Conservation and location of protein-protein interaction sites, modification sites, and DNA binding sites. (**C**) is a 180° rotation of (**B**) about the z-axis. (**A, B, C**) Sites are colored: DNA binding = green, Importin 7 binding = dark purple and dark green, Zn binding = purple, Karyopherin α5 binding = teal and orange, LEDGF binding = teal, Lysine acetylation = dark green, proline isomerization = orange, active site = royal blue, reverse transcriptase (RT) binding = brown.

To demonstrate different types of analysis supported by HIVToolbox, integrase (IN) was analyzed as a case study. IN is a well-studied multidomain and oligomeric viral protein that is essential for integrating viral DNA into the host genome, for viral infectivity, and for which potent inhibitors of its strand transfer function are chemotherapeutically available. Examples of how HIVToolbox can assist with hypothesis generation, experimental design, interpretation of results, and evaluation of structures and structural models are in **Figs. 1** **–** [Fig pone-0020122-g002] [Fig pone-0020122-g003] **4** **,** **Table 1**. One of the advantages is that data from many separate studies can be readily interpreted simultaneously. Several new hypotheses concerning IN complexes, DNA binding, nuclear import, and LEDGF binding are discussed. Since there is no structure of full length IN, these analyses also involved a number of different IN structural models that were generated by superposition of common regions in experimental IN structures (see **Methods**). This is an approach that was previously used by Wang et al. to propose a structure of the full IN monomer [8]. The models are available on the HIVToolbox website. We also used a recent structural model of the Prototypic Foamy Virus IN (PFV IN) to create a HIV-1 IN model, and analyzed this model with HIVToolbox [9].

**Figure 2 pone-0020122-g002:**
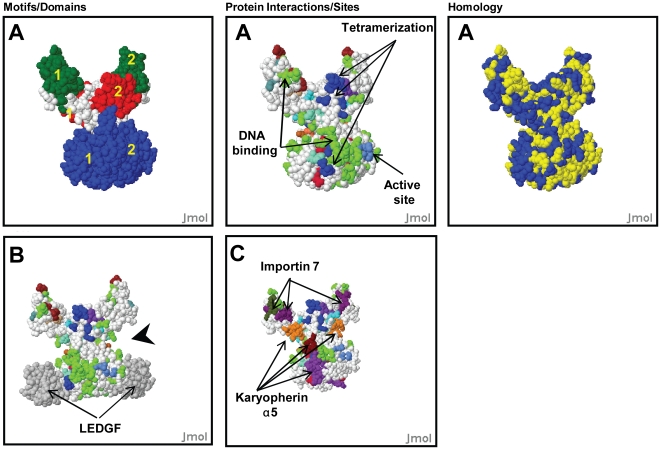
Analysis of Integrase model dimers hetero-tetramers. Output of HIVToolbox showing spatial positions on surface plots of different function sites on IN structural models in the same orientation. (**A**) Models of IN dimers showing domains (left panel), several functional sites (middle panel) and residues >98% conserved (right panel, yellow). In the left panel the yellow numbers indicate the monomer subunit for each domain. In the middle panel residues are colored as follows: DNA binding residues = green, tetramerization residues = blue, active site residues = royal blue (**B**) IN:LEDGF hetero-tetramer showing LEDGF domains (grey) and proposed viral LTR binding groove (arrowhead) (**C**) IN dimer showing nuclear import motifs.

**Figure 3 pone-0020122-g003:**
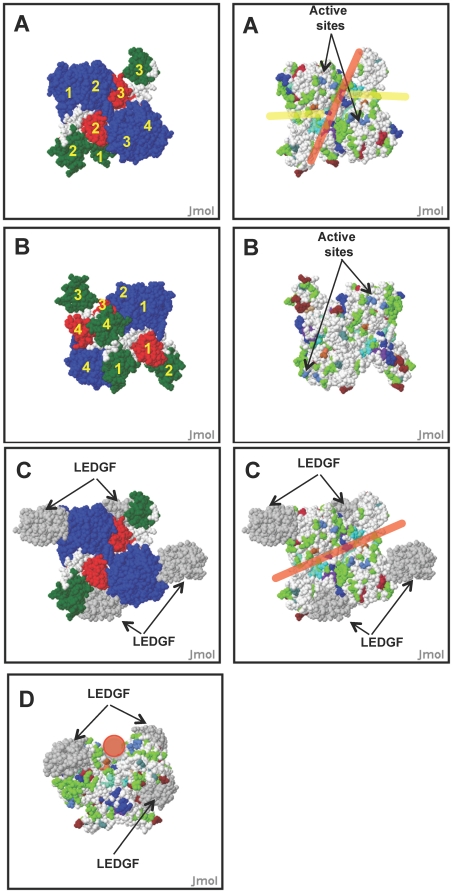
Analysis of Integrase model tetramers and hetero-octamers. Output of HIVToolbox showing surface plots of IN structural models. (**A, B**) IN tetramers showing domain organization (left panels) and locations of actives site residues (royal blue), proposed viral DNA binding grooves (yellow lines), proposed genomic DNA binding channel (red line), and zinc binding sites (cyan). Yellow numbers indicate the subunit too which the domain belongs. (**C**) IN:LEDGF hetero-octamers models showing organization of proteins (left panel) and proposed DNA binding groove (middle panel, red line). LEDGF subunits are colored grey. (**D**) An end-on view of the proposed host DNA binding channel in the IN:LEGDF hetero-octamer model shown in (**C**) (red circle).

**Figure 4:**
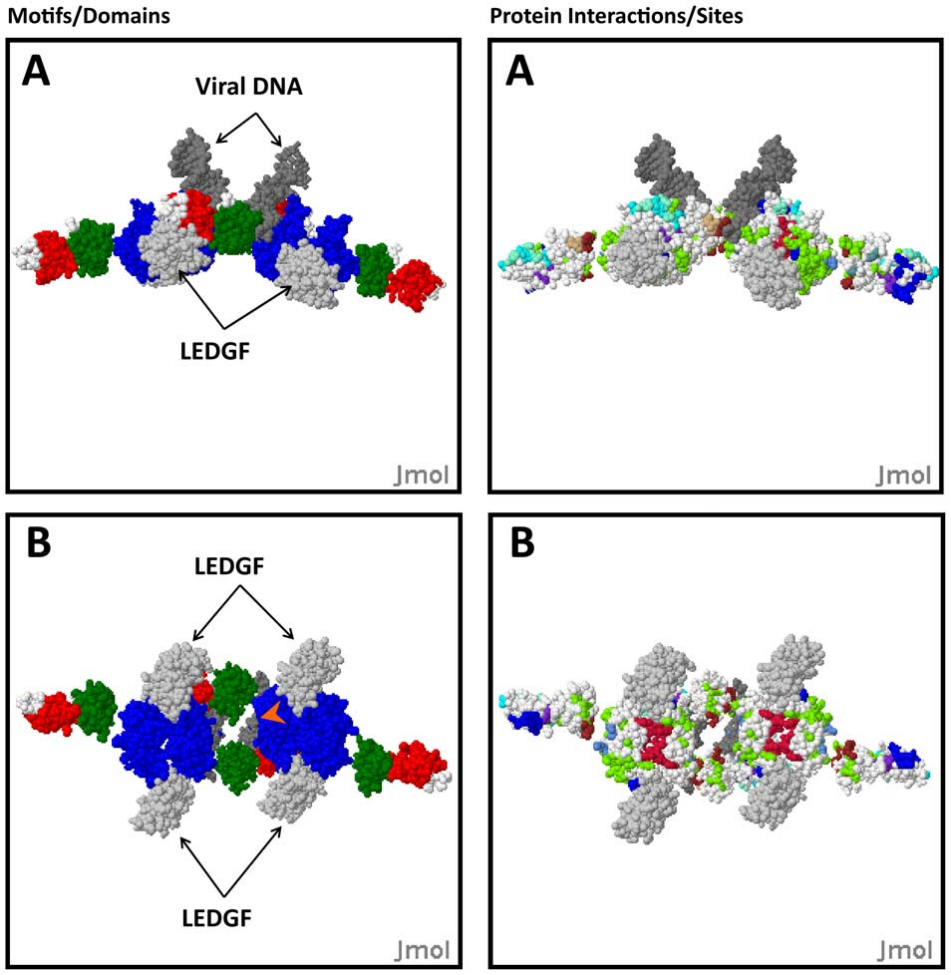
A model of IN:LEDGF:viral DNA based on the PFV IN structure. (**A**) A model of HIV-1 IN complex with 4 IN subunits, 4 LEDGF subunits (light gray), and two viral DNA strands (dark gray); (**A, B**) Left panels (IN NTD = green, CCD = blue, CTD = red. (**A, B**) Right panels show functional sites (green = DNA binding), red = dimerization interface in 1EX4 [10], cyan = dimerization interface in 1WJA [11]; blue = tetramerization interface in 1K6Y [8]; purple = zinc binding site [11]; brown = reverse transcriptase binding site [31], light brown = tetramerization residues [16]. (**B**) A 90° rotation about the Y-axis of **A**. Orange arrowhead indicated channel proposed to bind target DNA [36].

**Table 1:** Sequence conservation of CK2 sites in different strains of HIV-1.

CK2 sites in IN – [ST]xx[DE] – Domain		Site 1: 66–69 – THLE – catalytic	Site 2: 93–96 – TGGE – catalytic	Site 3: 195–198 – SAGE – catalytic	Site 4: 283–286 – SRQD – C-terminal
HIV-1/M (3787 isolates)	[ST ]	T -99%	T-99%	S-96%; C-1%; V-1%; T-2%	G-49%; S-49%; V-1%
	[DE]	G-1%; E-99%	E-96%; D-2%	K-1%; E-98%	V-1%; T-1%; D-94%; N-3%; E-1%
A (20 isolates)	[ST ]	T-100%	T-100%;	S-100%	G-81%; S-14%; D-5%
	[DE]	E-100%	E-100%	E-100%	D-95%; N-5%
A1 (149 Isolates)	[ST ]	T-99%; N-1%	T-99%; N-1%; L-1%	S-93%; V-1%; T-5%	G-71%; S-26%; M-2%; D-1%
	[DE]	S-1%; L-1%; E-98%; Q-1%	G-1%; T-1%; E-97%; Q-2%	E-98%; G-1%; C-1%	D-90%; N-6%; E-1%;
B (1443 Isolates)	[ST ]	T-99%	T-99%	S-96%; C-1%; T-1%	G-9%; **S-89%**; M-1%; R-1%
	[DE]	E-99%; G-1%	D-1%; E-98%	G-1%; D-1%; E-98%	**D-95%**; N-2%; E-1%
C (544 Isolates)	[ST ]	T-99%	T-99%	S-97%; C-1%; V-1%; T-1%	G-87%; S-9%; V-1%; D-2%
	[DE]	E-99%	G-1%; D-3%; E-96%	G-1%; K-1%; E-98%	G-1%; M-1%; D-96%; N-2%
D (85 Isolates)	[ST ]	T-100%	T-100%;	S-98%; T-1%	G-5%; **S-94%**;
	[DE]	G-2%; E-98%	D-1%; E-99%	E-99%; R-1%	**D-98%**; N-1%
F (3 Isolates)	[ST ]	T-100%	T-100%	S-100%	G-75%; S-25%
	[DE]	E-100%	E-100%	E-100%	D-75%; N-25%
F1 (39 Isolates)	[ST ]	T-98%; K-3%	T-98%; Y-3%	S-98%; I-3%	G-88%; S-10%
	[DE]	E-98%; W-3%	D-13%; E-88%;	T-3%; E-98%	D-90%; N-8%
F2 (8 Isolates)	[ST ]	T-100%	T-100%	S-100%	G-78%; S-11%
	[DE]	E-100%	E-100%	E-89%; *del-11%	D-100%
G (49 Isolates)	[ST ]	T-100%	T-100%	S-100%	G-88%; S-12%
	[DE]	E-100%	E-98%; Q-2%	E-100%	D-96%; E-4%
H (5 Isolates)	[ST ]	T-100%	T-100%	S-100%	G-50%; S-50%;
	[DE]	E-100%	E-100%	E-100%	E-100%;
J (6 Isolates)	[ST ]	T-71%; *del-29%	T-71%;*del-29%	A-29%; S-71%	G-57%; S-14%; P-14%; Q-14%;
	[DE]	D-29%; E-71%	E-71%; *del-29%	G-29%; E-71%;	G-14%; D-71%; Q-14%;
K (2 Isolates)	[ST ]	T-67%; R-33%	T-100%	S-67%; L-33%	G-33%; S-33%;
	[DE]	M-33%; E-67%	G-33%; E-67%	E-67%; Q-33%;	D-67%;

*‘del’ indicate that the residue is deleted or was not present in one or more structures.

Examination of the residues making contacts in different dimer structures of HIV-1 IN reveals that the relevant residues are largely clustered on one face of IN and are >98% conserved in 3787 IN sequences from the Los Alamos HIV Sequence Database (**Fig. 1A**) [8], [10], [11]. It is thought that dimeric IN is responsible for the 3′-processing activity in which IN removes 2 nucleotides from each end of the LTR in a reaction that primes a nucleoprotein complex (the intasome, the viral DNA genome and tetrameric IN) for the subsequent strand transfer reaction leading to integration and establishment of the proviral state [12]. When we examine the IN dimeric unit there are several features that are likely highly relevant to this activity.

A number of different studies have identified different sets of IN residues that bind DNA [10], [13]–[21]. Mapping all DNA binding residues onto the structures of IN shows a cluster of DNA binding residues near the active site (**Fig. 1C**). However, there are several other scattered clusters throughout IN. Comparison of the structure of the IN dimer shows that DNA binding residues in this binding groove continue into the juxtaposed catalytic domain of the dimer (**Fig. 2A**). The continuity of these additional DNA interacting residues (D207, K111, K136, E138, K215) [RefSeq: NP_705928] only becomes apparent in the dimer. In the IN:LEDGF hetero-tetramer model, LEDGF helps to define a putative DNA binding groove with a diameter of ∼25 Å, similar to the size of double stranded DNA (**Fig. 2B**, arrowheads). As previously observed a second cluster of DNA binding residues is located in the C-terminal domain (CTD) [14]–[17], [21]. Since the dimer is active in hydrolysis of the viral LTRs, these DNA binding residues are likely involved in binding the viral DNA as previously proposed in a number of models.

Analysis of IN with HIVToolbox also reveals that there is a striking overlap of clusters of DNA binding residues with several nuclear import motifs **(** **Figs. 1B, 1C** **,** **2C** **)**. Karyopherin α5 binds three regions on the surface of IN dimers [22], [23]. One of these sites overlaps almost entirely with the LEDGF binding site, whereas the other nuclear import sites overlap with DNA binding sites (**Figs. 1B, 1C** **,** **2C**), thus competition for these sites would be expected. Importin 7 binding requires two sites in the CTD; analysis with HIVToolbox reveals that these sites overlap with the cluster of residues that bind the viral LTR [24]. Consistent with the overlapping sites, the levels of viral genome are reduced >50% when the Importin 7 motifs are mutated [19]. However, analysis with HIVToolbox reveals that one of the Importin 7 sites overlaps with DNA binding residues. It is clear that the effect of karyopherins on binding of viral DNA needs to be considered in interpretation of their effects on nuclear import and binding LEDGF. This relationship becomes clear when HIVToolbox is used for interpretation. Nup153 is also implicated in nuclear import of IN, but its binding site within IN is not yet known [25].

The spatial arrangement of the nuclear import motifs on the surface of the IN dimer is striking. The five known nuclear import motifs are spatially contiguous like a ‘zipper; along the surface of the dimer, with two Karyopherin α5 sites located on one subunit, in trans with one Karyopherin α5 and two Importin 7 motifs on the other subunit (**Fig. 2C**). Some Karyopherin sites in these subunits are buried in the IN tetramer; however, two of the 5-motif zippers are located along the surface.

While Karyopherin α5 and Importin 7 both serve roles in nuclear import, they likely would compete with binding of IN to the HIV-1 LTR and to LEDGF. Presumably, these karyopherins would block these functional sites in the cytosol, but become activated after import of IN into the nucleus. It is not surprising given so many IN nuclear import motifs, which are likely redundant, that a recent re-evaluation found none to be required for nuclear import [26].

Summarizing these analyses with HIVToolbox suggests that it is important to determine 1) if LEDGF competes with Karyopherin α5 for binding IN, 2) if Importin 7 and/or Karyopherin α5 compete with binding of viral LTRs and 3) the stoichiometry of binding of IN dimers and tetramers with LEDGF and importins and how this is affected by DNA binding.

The tetramer of IN has strand exchange activity where the primed LTRs undergo a symmetric nucleophilic attack on the host DNA, integrating the viral DNA [8]. Reparation of the two 5 nucleotide gaps of host DNA flanking the proviral DNA insert and the two nucleotides of unjoined viral DNA left after a successful integration event is catalyzed *in vivo* by the host DNA repair machinery, restoring the integrity of cellular genome. Our IN tetramer model contains an asymmetric dimer of dimers, a symmetry evident from the 1K6Y structure [8]. The residues involved in tetramerization in 1K6Y are also well conserved in >97% of the IN sequences in HIVToolbox (**Figs. 1A, 1B**). The asymmetry of the tetramer is evident by comparing **Fig. 3A** with **3B**, which are flipped 180° with respect to each other. In **Fig. 3A**, the two active sites are on opposing sides of a channel lined with some DNA binding residues and separated by ∼17 Å along the channel, perfectly spaced to excise a 5 bp fragment of DNA, the known product of the strand exchange reaction. The two grooves in the dimer proposed to bind the viral LTRs (**Fig. 3A** **, yellow line**) are aligned nearly perpendicular to proposed DNA binding channel. When this molecule is flipped (**Fig. 3B**) the other two active sites in the tetramer can be seen and are separated by 74 Å; there is no obvious channel that could accommodate the host DNA.

LEDGF binds to IN and is known to play a role in selecting sites of integration in the human genome [27]. To explore this interaction, an IN:LEDGF hetero-octamer model was generated by superposition of the CCD domain of IN in the LEDGF:IN complex with the CCD domains of IN in our model tetramer as done for other IN models [8], [28]. In addition to lining the groove in the dimer (**Fig. 2B**), LEDGF also extends the DNA binding channel proposed to bind the host DNA (**Fig. 3C, 3D**). Although LEDGF is not critical for the integration reaction, positively charged LEDGF residues R404, R405, K407, and K424 in LEDGF line the proposed DNA binding channel and may play a role in chromosomal site selection in the host genome, consistent with its known effects on selectivity for the site of integration [27]. The LEDGF binding site in IN is highly conserved with the exception of D167, which has a conservative substitution of E in 19% of IN sequence [29], [30]. Alternatively, this high sequence conservation could also be due in part to the binding site for Karyopherin α5, which overlaps with the binding site of LEDGF.

The model proposed by Faure et al. suggests that IN dimers bind LTRs and that dimerization of these dimers brings the viral genome ends together and allows binding of the tetramer to the host genome for initiation of the strand transfer exchange [8]. In this model, the control of dimer tetramerization may be an important aspect of IN function. By using HIVToolbox, we can look for other IN elements that overlap with the residues that are involved in the tetramer interface to generate new hypotheses. HIV-1 reverse transcriptase binds to the CTD of IN, a region that is juxtaposed to L241 and L242, residues known to block tetramerization [16], [31]. K258, a residue that binds reverse transcriptase, is also acetylated and thus could be involved in controlling the release of RT and the multimerization state of IN [8], [32]. Collectively, these analyses reveal the power of HIVToolbox in generating new hypotheses, evaluating structural models, and interpreting experiments for a well-studied protein.

### Analysis of a HIV-1 IN model based upon the Prototypical Foamy Virus integrase structural model

The Prototypic Foamy Virus (PFV) IN model with viral DNA is based on the structure of a 3 domain IN PFV monomer dimerized with an IN catalytic domain and bound to viral DNA [33]. We used this structure to build a model of the HIV-1 IN tetramer bound to viral DNA [33]. The PFV model is gaining acceptance among scientists, but there are a number of issues that need to be reconciled if this is indeed a structural architecture that is representative of HIV-1 IN: 1) The center channel that is proposed to bind the target DNA is only 12–13 Å wide at some points (e.g. R231-R231) and the double helix of the target DNA has a diameter of 20 Å. Furthermore, the linker between the CTD and CCD of HIV-1 IN is 6 residues shorter than in the PFV IN. Considering that the CTD linker is a fully extended helical conformation in the structure of the PFV IN, and that this extended conformation of the CTD is important for forming the central DNA binding channel in the PFV IN tetramer, it does not seem likely that the domains of HIV-1 could assume this structural configuration.

We generated a tetramer model of HIV-1 IN with two viral DNA fragments, by superposition of the HIV-1 and PFV domains and superposition of the structure of the LEDGF:IN complex; interdomain linkers were ignored in this model (see Methods; **Fig. 4**). Analysis of the PFV-based IN model with HIVToolbox also shows: Only one of many DNA binding residues in HIV-1 IN maps to the viral DNA binding site or the proposed DNA binding channel in PFV IN (**Fig. 4A, 4B**, right panels). The LEDGF binding site in this model is not positioned where it could make contacts with the target DNA. Further analysis with HIVToolbox shows that the LEDGF HIV-1 IN binding site (residues 161–174) is highly conserved in greater than >97% of the 3787 viral sequences in HIVToolbox with the exception of D167 (which has a conservative substitution of E), and K173 (which has a conservative substitution of R). This site is not conserved in PFV IN (residues 250–263 in HFV IN) [UniProt: P14350]. Considering the 3D location of the analogous LEDGF binding site in HIV-1 IN and that PFV IN does not bind LEDGF, it is difficult to envision how LEDGF could affect the sites of HIV-1 DNA integration if IN is structured as in the PFV-based model [9], [30], [34], [35].

Based on these observations we must consider the possibility that while the integration reaction is conserved, the oligomeric structures of the catalysts may differ between HIV-1 and HPV. The TN5 transposase has a different tetramer structure and the PFV IN and may also have a structure of its active tetramer that is distinct from HIV-1 IN [36], [37]. However, since Raltegravir and Eltegravir both bind PFV Integrase and block strand transfer, it is likely that the binding of the viral DNA ends in both PFV and HIV-1 INs are similar [36].

Alternatively, differences in the HIV-1 and PFV structures may represent differences between those states required for completion of the IN reactional set. Our new model is based on the apo form lacking DNA, while the HFV model in complex with viral DNA. There are also several possible intermediates in the strand transfer reaction. None of the existing models, including ours, is consistent with the recently determined low-resolution electron microscopy (EM) tomography image of the HIV-1 IN tetramer: DNA complex [38]. This could again reflect that the EM images were of IN tetramers bound to a strand exchange intermediate different than the aforementioned models. While we have not assessed a number of previous IN models, these models can be readily added to HIVToolbox for relational assessment. This analysis shows how useful HIVToolbox is in evaluating structural models.

### Integrase is phosphorylated by Casein Kinase 2 (CK2)

**Figure 5:**
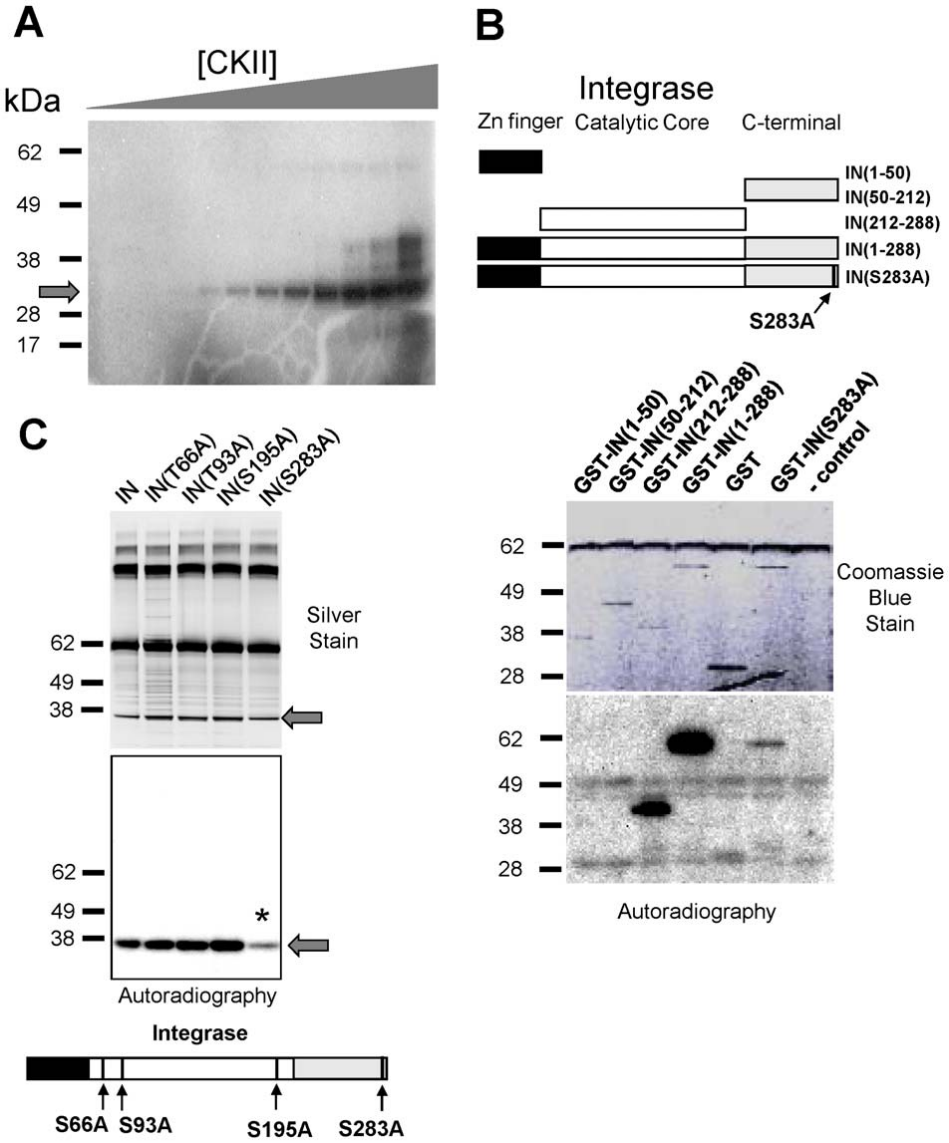
Integrase is phosphorylated by CKII on serine 283. (**A**) Phosphorylation of IN by CK2. Reactions containing IN, ^32^P-γ-ATP and CK2 were serially diluted 1∶3 starting from 50 U/mL (right lane). The extent of phosphorylation was visualized by SDS-PAGE and autoradiography. (**B**) Phosphorylation of IN domains by CK2. Reactions as in A and contained purified GST-IN or GST-IN domains as indicated in the diagram. IN(S283A) is a point mutation of one of the putative CKII phosphorylation sites. (**C**) His-tagged point mutants of putative CKII sites within IN were phosphorylated by CK2 as in (A). A control reaction lacking IN showed no phosphorylation (not shown). Reduced phosphorylation of S283A mutant is indicated (*). Samples were normalized for protein concentration.

Since HIVToolbox maps the ∼5000 minimotifs from Minimotif Miner, new functional elements in HIV-1 proteins can be identified [10], [11]. CK2 has been reported to phosphorylate HIV-1 matrix, Rev, and Vpu proteins *in vitro*, but is not known to phosphorylate IN [13], [39], [40]. Other labs have predicted minimotifs in HIV-1 proteins [41]–[43]. IN contains four putative CK2 consensus phosphorylation sites (T66, T93, S195, S283) [RefSeq: NP_705928]. Phosphorylation assays of recombinant His-tagged IN were performed using commercially obtained purified recombinant human CK2 (New England Biolabs) in the presence of ^32^P-γ-ATP. Serial dilution of CK2 showed phosphorylation of recombinant HIV-1 IN substrate with as little as 0.02 U/mL of kinase (**Fig. 5A**).

We next examined which IN domains were phosphorylated by CK2. Recombinant IN and individual domains fused with GST were purified and subjected to CK2 phosphorylation assays. The full length IN(1–288) gave the strongest signal for phosphorylation when compared to N-terminal domain [NTD, IN (1–50)] and the catalytic core domain [CCD, IN (1–212)], both of which were weakly phosphorylated (**Fig. 5B**). IN (212–288) CTD was phosphorylated to a level comparable to that of the full-length IN (1–288). Reactions with control GST or lacking substrate (- control) showed no significant phosphorylation. These domain-mapping experiments indicate that IN CTD is the primary target of CK2 phosphorylation.

To further investigate the sites of IN CK2-mediated phosphorylation, we generated all possible permutations of Ser/Thr to Ala point mutations in the 4 putative CK2 phosphorylation sites (T66, T93, S195, S283). His-tagged IN and these mutants were purified, normalized for their protein content, and tested in kinase reactions using purified CK2. The S283A substitution severely compromised CK2 phosphorylation, reducing it by 90% when compared to wild type IN, whereas other mutants were without effect (**Fig. 5B, 5C**). Phosphorylation of S283 was also reflected in an analysis of all double, triple, and quadruple mutants for the potential CK2 phosphorylation sites (data not shown). Only those mutants which had a S283A mutation showed reduced phosphorylation of IN. When the S283A mutation was introduced into the IN (212–288) CTD fusion protein, this also reduced the majority of IN phosphorylation when compared to full length IN (**Fig. 5B**). The data indicate that S283 is the principal *in vitro* CK2 phosphorylation site in IN. Despite efficient phosphorylation of S283 *in vitro*, mutation of this phosphorylation site (S283A) in a recombinant virus showed no impairment of IN nuclear import, syncytia formation, or detectable accumulation of p24 when assayed for growth through immortalized cell lines (data not shown), nor when the C-terminal amino acids containing the CK2 consensus minimotif (275–288) were deleted [44] (data not shown).

### Interpretation of CK2 phosphorylation sites in integrase

Since one purpose of HIVToolbox is to assist with interpretation, the CK2 sites within IN are discussed within this context. The CK2 site at 283–286 was phosphorylated by CK2. These residues were poorly conserved in the IN sequences of 3787 isolates, as determined with a position-specific scoring matrix (PSSM) in HIVToolbox. However, when we examined conservation in different strains, IN 283–288 was highly conserved in >1500 viruses from Group M, clades B and D (**Table 1**). One interesting observation was that the CK2 consensus sites that were not phosphorylated were highly conserved. The group/clade analysis of the CK2 phosphorylation sites in HIV-1 IN can be performed for any sequence element of interest by using the strain selection function and sequence alignment section in HIVToolbox. A more complete description on interpretation of these CK2 sites in different HIV-1 groups and clades is shown in **Table 1**.

Examination of the spatial relationship of this CK2 phosphorylation site to other functional IN regions shows this site is in an intrinsically unstructured segment on the C-terminus extending 18 residues from D270, the last structured residue in 1EX4. In the structure of the IN monomer the phosphorylation site would be expected to lie away from the active site and likely only have access to some lysine acetylation sites and some of the DNA interacting residues [32], [44]–[46], which is in agreement with its lack of impact on viral replication upon deletion (unpublished data, M. A. Muesing). However, it is noteworthy that in our IN tetramer model the disordered fragment containing the CK2 site is well positioned to sit in the channel that is lined with DNA binding residues and is in proximity to the IN active site in *trans* in other subunits. One possibility is that this unstructured region is not disordered in the tetramer and blocks the DNA binding channel before IN is transported into the nuclear compartment. This may help to repress access to other molecules, but would likely be dispensable in assays that assess replication, as was previously observed [44] (data not shown). Alternatively, this CK2 site might have some functional role in a select set of virus/host interactions, as it seems like the site is selected against except in the case of M/B and M/D viruses. The surrounding residues in the unstructured region are >93% conserved in the 3787 isolates, whereas only the consensus residues for the CK2 site are poorly conserved. Since the coding sequence for IN residues 271–288 overlaps with the code for amino acids 1–18 of the Vif amino-terminus, the consequence of these observations should also be considered with respect to Vif functionality. However, regardless of the identity of the amino acid specified at IN 283 (either serine or glycine), the corresponding amino acids in Vif (Q12/V13) are invariant in all HIV-1 clades.

The other CK2 consensus sites at 66–69, 93–96, and 195–198 were highly conserved in >98% of 3787 isolates, sometimes having conservative D/E or S/T substitutions that conserve the [ST]xx[DE] minimotif (**Table 1**). These sites were not significantly phosphorylated *in vitro*. Examination of IN structures showed that these sites are on the surface of the monomeric unit (**Fig. 1A**). The sites at 66–69 and 93–96 are also on the surface of both experimental [8], [10] and our model structures of IN (**Fig. 1A**); however, the site at 195–198, is buried in model dimers and tetramers. Thus, it is conceivable that this site may not have been phosphorylated *in vitro*, if the assay contained multimerized IN, and we cannot thus rule out that IN monomers may also be phosphorylated at this position.

### Design and Implementation

#### HIVToolbox model and database construction

**Figure 6:**
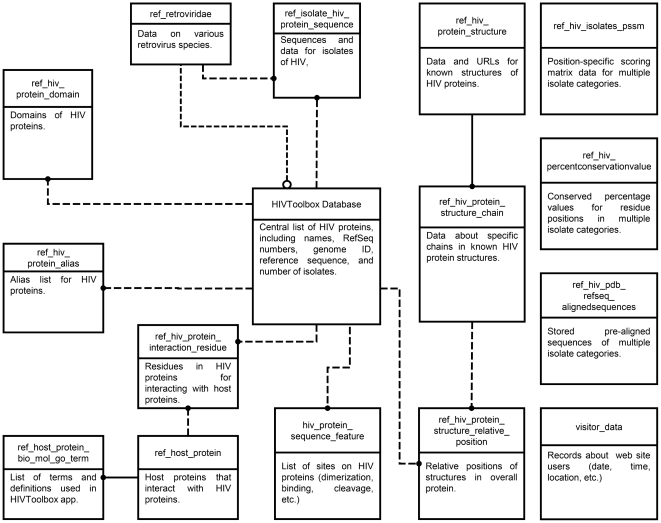
Entity Relationship diagram of HIVToolbox model as a MySQL database. Boxes show different tables (titles listed on top row) and a description. Foreign keys between tables are shown.

The knowledge domain we sought to model was that of HIV-1 proteins, including sequences, structures, functions, and functional interactions with other small ligands and macromolecules. We generated a model for sequence, structure, and function of HIV-1 proteins. This model includes RefSeq and isolate sequences for the 24 HIV-1 proteins, strain classifications, protein structures, protein subcellular localizations, virus-host protein interactions, requisite host proteins, and the cellular and molecular functions of each viral protein and its associated host proteins (**Fig. 6**).

HIVToolbox was built as a MySQL database importing and integrating data from existing databases, with the data sources shown in **Table 2**. Integration of these data required a number of manual and computational annotation efforts, as well as computational processing of some of the data into new tables as follows: The publications for all tuples of the HIV-1 protein-protein interaction database were reviewed to identify and re-annotate the interacting residues on the HIV-1 protein in those entries where this information was known. Alias lists for HIV-1 proteins were generated from databases and the primary literature. Minimotifs from the primary literature were annotated to identify experimentally confirmed HIV-1 minimotifs. Several models of HIV-1 proteins were created by superposition of atoms in structures that had common regions. Data for host proteins that are required for the HIV-1 life cycle were annotated from several RNAi screens reported in the primary literature. Functional sites in HIV-1 proteins for interaction with other HIV-1 proteins, other macromolecules, metals, etc., as well as localization of HIV-1 proteins were annotated from both external databases and the primary literature. Sequence alignments of all HIV-1 protein isolate sequences in the database were performed for each HIV-1 protein, as well as for different groups and clades. These alignments were used to generate position specific scoring matrices that are stored in the HIVToolbox database. Variable sequence numbering frames used for RefSeq sequences and PDB sequences were computationally matched and stored in the database. Statistics for the data in the HIVToolbox database are summarized in **Table 3**. Parsers were built for all imported databases and can be used to frequently update the data in HIVToolbox.

**Table 2:** Sources of data in the HIVToolbox MySQL database.

Table Name	Data Type	Source
hiv_protein_annotation	Annotations for HIV-1 proteins and effects on host proteins	NCBI/PubMed; NCBI/Protein [6]
hiv_protein_sequence_conservation	List of each residue in HIV-1 proteins and associated conservation in existing data	Self-generated from NCBI/RefSeq data [6]
hiv_protein_sequence_feature	List of interesting sites on HIV-1 proteins (sites for dimerization, binding, cleavage, etc.)	NCBI/PubMed; HIV PPI database; *RCSB/PDB [3], [6], [56]
ref_group	HIV-1 groups and accession numbers	NCBI/RefSeq [6]
ref_hiv_isolates_pssm	Position-specific scoring matrix data generated by ClustalW from HIV-1 isolate sequences.	Calculated from Los Alamos/HIV and ; NCBI/PubMed [6], [10]
ref_hiv_protein_alias	HIV-1 proteins and synonym names	NCBI/PubMed [5]
ref_hiv_protein_domain	List of domains in each HIV-1 protein and location of each domain in its protein	NCBI/Conserved Domains [5]
ref_hiv_protein_interaction_residue	List of interaction sites for HIV-1 proteins and residue positions for each interaction site	NCBI/PubMed; HIV PPI database [4], [5]
ref_hiv_protein_structure	List of structures in HIV-1 proteins and data about each structure	RCSB/PDB [56]
ref_hiv_protein_structure_chain	Sequence information for structures of HIV-1 proteins	RCSB/PDB [56]
ref_hiv_protein_structure_relative_position	Positional information about structures of HIV-1 proteins	NCBI/RefSeq; RCSB/PDB [5], [56]
ref_host_protein	List of HIV-1 host proteins, their sequences, and whether or not the protein is required for HIV-1 replication	NCBI/RefSeq; Literature [5], [57]–[60]
ref_host_protein_bio_mol_go_term	List of term types used in HIV-1 databases	GeneOntology AmiGO [33], [61]
ref_isolate_hiv_protein_sequence	List of HIV-1 isolates, their sequences, accession numbers, date and country of infection, patient codes, and source database code	NCBI/Protein; Los Alamos/HIV [4], [5]
ref_retroviridae	List of retroviruses, accession numbers, and links to articles	NCBI/Taxonomy [5]
ref_subtype	List of subtypes of HIV-1 and associated group of subtype	NCBI/Taxonomy [5]
ref_swissprot	List of Swissprot IDs and associated gene symbols	UniProt/UniProtKB [62]
ref_swissprot_pdb	List of PDB ID's and corresponding Swissprot IDs	UniProt/UniProtKB; RCSB/PDB [56], [62]
MnM database	Predicted minimotifs	Minimotif Miner [10], [11]

*Sequence features that are multimerization interfaces were calculated in Molmol based on residues that were less than 3.25 Å away from at least one residue in another subunit [63].

**Table 3:** Statistics for data in the HIVToolbox database.

Data type	Number
HIV-1 proteins	24
HIV-1 residues	3137
HIV-1 protein isolate sequences	203,810
HIV-1 protein-protein interactions	313
HIV-1 experimental structures	621
HIV-1 experimental structure chains	1,356
HIV-1 model structures	6
HIV-1 model chains	34
HIV-1 protein domains	49
HIV-1 putative motifs	5,312
Experimentally determined motifs	198
Host proteins	2,096
Required host proteins	755
HIV-1 protein functional elements mapped	560
HIV-1 isolates with homology data	153,000
HIV-1 position specific-scoring matrices	104

#### Construction of HIVToolbox web application

**Figure 7:**
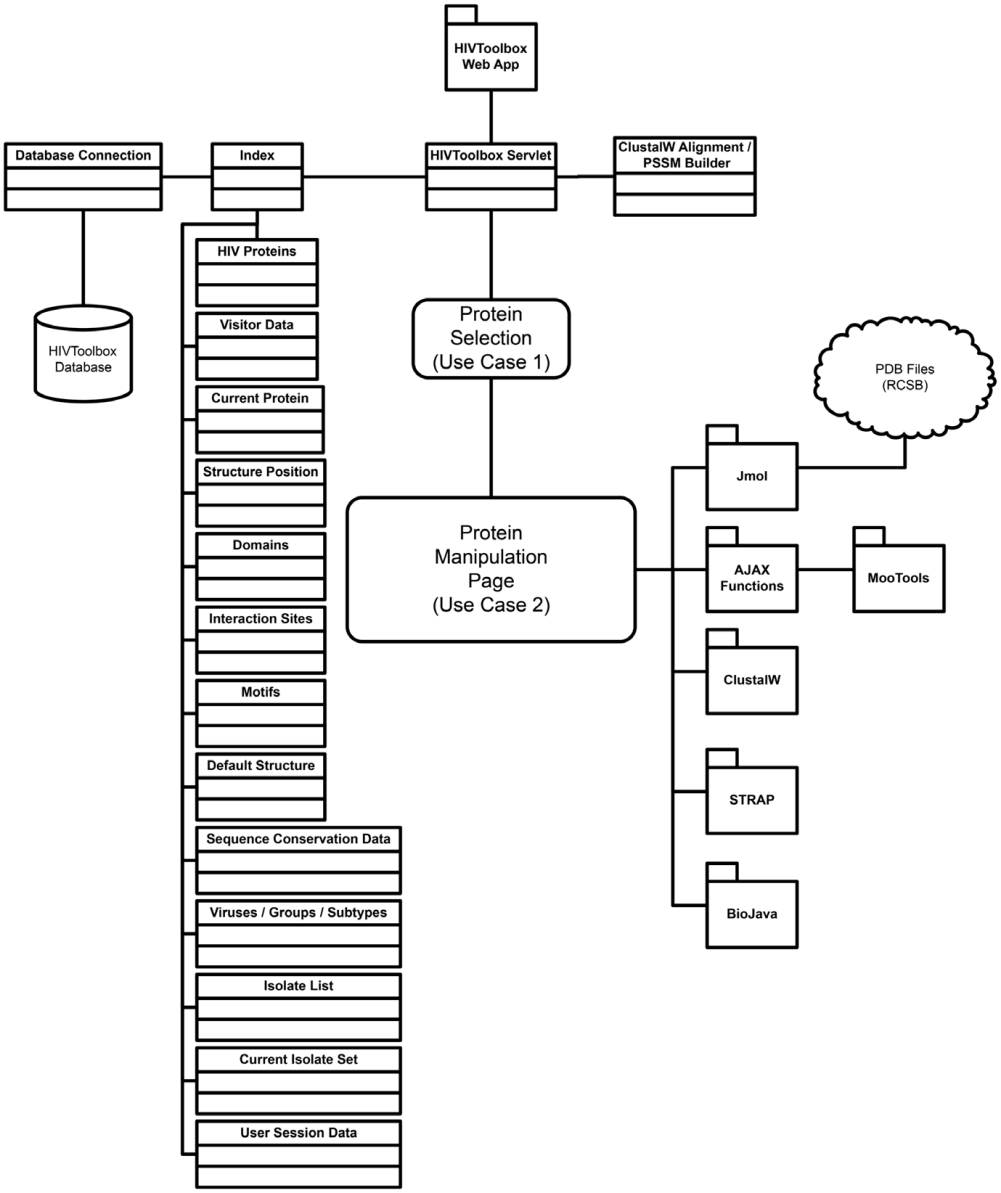
Diagram of software architecture of HIVToolbox.

HIVToolbox is a web-based application built as a Java 2 Enterprise Edition servlet that pulls data from a server-side MySQL relational database. The application retrieves data from tables of the database (**Fig. 6**) and stores the data in a number of “beans”, Java objects that correspond to query results of data in the tables of the database. Beans are easily stored and retrieved by the application. The majority of the application's data processing and calculations are performed on the server to minimize time transmitting data over the internet to the end user. The overall architecture of HIVToolbox is shown in **Fig. 7**.

HIVToolbox incorporates the results of several open-source utilities. Java 2 Enterprise Edition [http://www.java.com], an open-source web application development tool and language, provides the framework of the application. MySQL [http://www.mysql.com], an open-source relational database system, is used to store and retrieve the considerable data that are required to run HIVToolbox. Apache Tomcat [http://tomcat.apache.com], an open-source web application server, is used to serve HIVToolbox. Jmol [http://www.jmol.org], an open-source Java molecular viewer, is used to view the protein structures. MooTools [http://mootools.net], an open-source JavaScript library, powers the conservation slider. ClustalW [http://www.clustal.org], an open-source sequence alignment utility, provides the basic algorithm used to align sets of protein sequences and create the PSSMs [47]. BioJava [http://www.biojava.org], an open-source Java-based biology library, is used to properly format amino acid sequences. STRAP [http://www.bioinformatics.org/strap] is used for formatting aligned protein sequences [48].

Finally, Minimotif Miner [http://minimotifminer.bio-toolkit.com] is used to provide the minimotif data displayed in HIVToolbox [49], [50]. To calculate minimotif probabilities we implemented a previously published algorithm [51]. Since the minimotifs displayed in the sequence windows are predictions based on conserved consensus sequences and instances, the Minimotif Log window displays a probability that the predictions are correct. This calculated probability is based on a portion of the *sig* score algorithm [51]. Briefly, the probability ***p*** **_1+_** that a given motif will occur at least once in a protein is calculated using the formula:
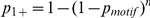
where *n* is the number of positions in the protein where the given motif can occur, and *p_motif_* is the probability of a particular motif occurring at any given position in the protein. Further details on the calculations of the ***p*** **_1_**_***+***_ value and the *sig* score algorithm can be found in Davey et al [51]. It should be noted that the frequencies of amino acid occurrences were derived from pre-calculated values based on the human and HIV-1 proteomes; probability values based on both proteomes are shown in the application. Calculations based on the human proteome values were included since HIV-1 must infect a human host cell to replicate, and thus it may be useful to compare the probabilities derived from both sets of amino acid frequencies.

#### User Interface and workflows

HIVToolbox processes and presents this data in an easy-to-use open-access web application (accessible at HIVToolbox [http://hivtoolbox.bio-toolkit.com]). HIVToolbox was implemented as a website in order to minimize usage barriers; a standalone application requiring downloading and installation would discourage many prospective users from trying HIVToolbox. In designing the application, we considered common analysis workflows for investigating proteins. For example, new functional regions can be identified by plotting sequence conservation onto protein structure surfaces using tools such as VENN, ConSurf, and Evolutionary Trace [52]–[54]. A unified interactive view of protein sequence, structure, and function was built and a schematic representation of the software architecture is provided in **Fig. 7**.

**Figure 8:**
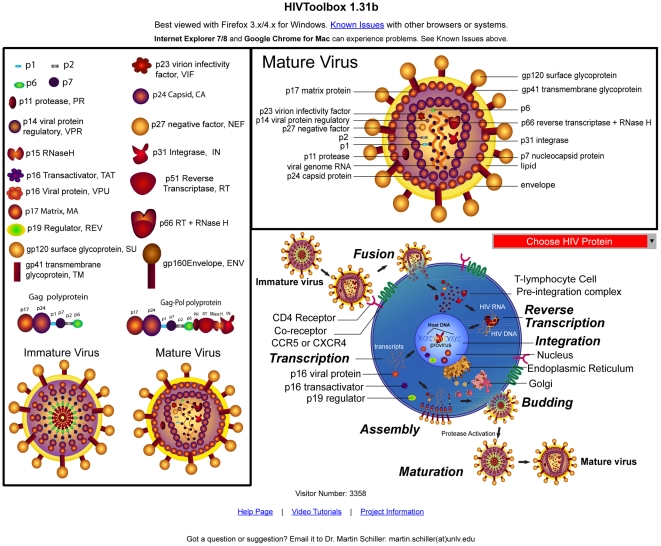
Protein selection page of the HIVToolbox website. An image of the Introduction page of HIVToolbox is shown. Selection of any HIV-1 protein name or protein object launches an interactive results page about the protein. A drop-down menu is provided for non-graphical access to proteins.

**Figure 9:**
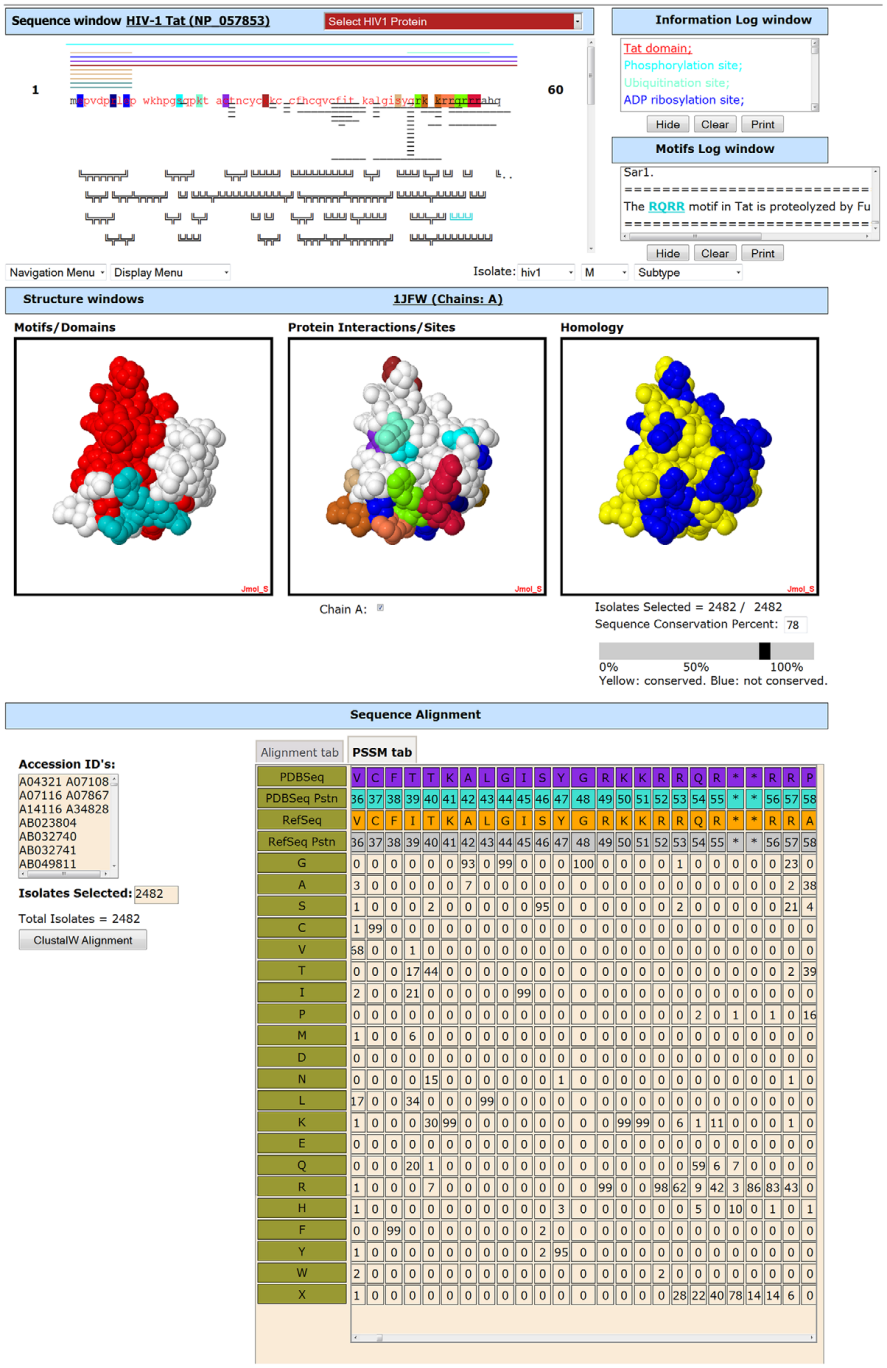
Interactive protein display page for Tat in HIVToolbox. Sequence window, Structure windows, Log windows, and Sequence Alignment section of HIVToolbox are shown. The interactive results page for HIV-1 tat is shown. The scrollable sequence window shows the protein sequence, domains (with colored fonts), functional residues (highlighted), protein-protein interaction sites (thin lines under sequence), mapped protein structures (thin colored lines over sequence) and minimotifs (figures under sequence). The synchronized interactive structural displays show domains and selected minimotifs (left panel), functional sites and selected protein-protein interaction sites (center panel), and residues conserved at or above a sequence conservation threshold selected with a slider or text box (right panel). The Sequence Alignment section shows alignment of a representative set of 20 sequences with the RefSeq sequence and the structure sequence. A second tab reveals a position specific-scoring matrix of amino acid frequencies at each position in the protein. More details about the features and use of HIVToolbox are in the supplement, and video tutorials are at Bio-Toolkit [http://www.bio-toolkit.com].

The basic workflow for HIVToolbox is as follows: At the application's introductory webpage, users can select the HIV-1 protein they wish to investigate from a diagram of the HIV-1 life cycle (**Fig. 8**). The application then displays the primary interface, an interactive console from which the user can perform a variety of functions related to the sequences, structures, and functions of the selected protein (**Fig. 9**). Alternatively, the primary interface page can be accessed directly via links in the HIV-1 protein structure pages at the Protein Data Bank, which pre-loads the selected structure.

The HIVToolbox viewer has four main integrated components: a sequence viewer, an array of structural displays, two log windows, and a sequence alignment section. Each window/section/menu has a number of interactive features that trigger coupled events in other application components. The sequence viewer shows an HIV protein sequence that was selected from the introduction page or navigation menu bar. The interaction of the user with the program is shown in **Fig. 7**. Help pages and video tutorials can be accessed by selecting the HIVToolbox icon at bio-toolkit.com.

The main interface is shown in **Fig. 9**.The four main sections are:

**1.** The **Sequence Window** displays the amino acid sequence for the primary structure of the HIV-1 protein using IUPAC single-letter amino acid abbreviations [55]. The sequence letters are color-coded to indicate known domains in the protein, and highlighted sequences indicate known functional sites such as crucial residues for enzymatic activities, binding sites, multimerization interfaces, and post-translational modifications. Sequence regions for those structures that have been determined are shown as a series of colored lines above the sequence; all known structures for each HIV-1 protein are mapped in this manner. Hovering the mouse pointer over any element displays metadata in a popup balloon.

Selecting a structure line loads the associated structure into three structure windows (discussed below); model structures are indicated by dotted lines. Directly under the protein sequence, horizontal thin black lines mark known host protein interaction sites. Clicking a line causes the interaction site to be colored in the appropriate structure window. Under the protein interaction site lines are a series of figures representing putative minimotifs predicted from consensus sequences or instances in the Minimotif Miner database [http://minimotifminer.bio-toolkit.com] [49], [50]. Selecting one of these figures colors it in both the sequence and structure windows. Any number of minimotifs can be selected at the same time. Selection of any of the features in the sequence window loads an associated hyperlink to the log windows and loads or colors the selected feature in one of the structure windows.

**2.** The **Structure Windows** are comprised of three distinct interactive three-dimensional structures of the HIV-1 protein loaded in the Jmol molecular viewer, a 3D structural viewer that runs as an applet within HIVToolbox. Each window displays an interactive structure that can be zoomed or rotated and chains of the structure can be selected for viewing from a set of checkboxes. Several standard Jmol structure analysis functions are accessible through a mouse right click. Hovering the mouse over any part of the structure reveals the residue and its number in the sequence. The three windows are synchronized so that the user is always viewing the same portion and orientation of the structure with respect to the other two structure windows.

Each window shows different features that can be visually compared: The Motifs/Domains window displays minimotifs and domains of the loaded structure, coloring the sections of the structure corresponding to the selected minimotifs and domains appropriately. Linker regions are colored white. The Protein Interactions/Sites window displays the functional sites colored similarly to the highlighted positions in the sequence window. Selection of any of the host-HIV-1 protein interaction sites colors these residues in this window.

It is useful to visualize sequence conservation on protein structure surfaces using tools such as VENN, ConSurf, and Evolutionary Trace [52]–[54]. This function is implemented in the Homology Structure window, which consists of a Jmol window and a control panel directly beneath the window. The window itself shows the conservation of the residues of the loaded structure. The control panel beneath the window consists of a slider and a text input box, allowing the user to select a desired conservation percentage threshold. Initially, all residues are colored yellow, indicating that they are all conserved, being above the 0% conservation threshold. As the user moves the slider or changes the percentage directly, residues that are not conserved in at least the selected percentage of the isolates on record are changed to a blue color; the number of isolates used for the calculation is shown. The isolates selected can be interactively changed based on species, group, and subtype selected from a navigation menu. This display enables visual correlation of residue conservation with putative minimotifs or other functional sites, enabling prediction of important functional minimotifs in the virus. The Alignment Section (described below) of HIVToolbox is tightly coupled with this viewer.

**3.** The Information Windows consist of the Information Log and Minimotif Log windows. The Information Log initially shows a list of the domains and interaction sites the protein is known to have; each feature is displayed in the color corresponding to that particular feature in the Sequence and Structure windows. As the user interacts with the Sequence Window (e.g., selects structures, motifs, or interaction sites), the selections and colors representing the selected elements are loaded into the Information Log. Further information about each feature can be obtained by clicking on its hyperlinked listing in the Information Log, taking the user to a primary literature source for that feature. Additionally, the Information Log can be hidden, and its contents can be cleared or printed. The Minimotif Log displays information about the putative minimotifs that have been found in the current protein; when the user selects a minimotif in the Sequence Window, information about the minimotif is loaded to the Minimotif Log. Selection of hyperlinked minimotifs reveals additional information in the Minimotif Log or opens a new browser with the primary source for the minimotif.

**4.** The Sequence Alignment section consists of a window with two tabs, the Alignment Tab and the PSSM Tab. The user selects a set of isolates to work with by using three navigation pull-down menus to select the species (HIV-1 or HIV-2), group (M, O, etc.), and subtype (A, B, B/A recombinant, etc.) of the isolates in the database. A display panel on the left shows a list of accession numbers for each isolate in the selected set. It displays the total number of isolates in the HIVToolbox database that match the selected criteria. Clicking the “ClustalW Alignment” button after selecting a set of isolates, retrieves a stored alignment calculated via ClustalW, and a selection of aligned isolates are displayed in the Alignment Tab [47]. The alignment of 20 randomly selected isolates with the RefSeq and PDB sequences are shown. Importantly, an alignment of the PDB sequence with the RefSeq sequence with residue numbers allows direct comparison of sequence and structural information, a limitation often faced by biologists in routine experimental design and interpretation. A calculated PSSM is available in the PSSM Tab for the selected isolate set. In addition, the residues that are conserved above the current percentage threshold selected in the Homology Structure window's slider are colored yellow in the structure window and also highlighted in yellow in the PSSM display. An image of the Sequence Alignment section is shown in **Fig. 9**.

## Discussion

While several HIV databases have played a central role in forwarding HIV research, these sources have some disadvantages. The data is spread out among numerous sources, each providing a different search interface with its own syntax, restrictions, and output options. With the information spread out among multiple sites, the results needed to address specific questions must often be reformatted and pieced together, a barrier that discourages investigation of many questions. Queries to these databases generally return tables as results or have focused user interfaces that can address a limited set of questions.

HIVToolbox rectifies these problems by consolidating relevant information about HIV into one location, and presenting the information to the end user in a single window consisting of an easy-to-use graphical interface. This integration does not come without a cost. The principle disadvantage of the integrated and unified databases is that each individual domain of knowledge is better maintained by a group of focused experts. Furthermore, additional union tables are required for cross-referencing information retrieved from different databases. To address these limitations, we rely on the domain experts of the individual projects, and have built parsers and data miners that can be used to keep the data current in HIVToolbox. Although not a perfect solution, this methodology allows centralization of data while keeping the data current.

We have shown the utility of HIVToolbox in experimental design and interpretation by analyzing IN. HIV-1 has a very complicated intracellular life and it is difficult to resolve its functional pathways without an integrated tool such as HIVToolbox that brings all of the data together in a common user-interface. HIVToolbox allows virologists to use structural information in their experimentation and structural biologists to have easy access to functional information. Questions of interest that would have been virtually impossible to discern using the existing data management are now readily apparent and addressable. We see no reason why HIVToolbox could not be used to study the other HIV proteins and this integrative approach should be readily adaptable to the study of more complex biological systems.

### Availability and Future Directions

HIVToolbox is open access and can be found at http://hivtoolbox.bio-toolkit.com. HIVToolbox can also be accessed through links of HIV structures in the Protein Data Bank. The application is platform independent, written in Java 2 Enterprise Edition, JavaScript. The application is tested and supported in Firefox 3.0 or higher. Other requirements include Java Runtime Environment 1.6 or compatible browser plug-in capable of running Java applets. Other browser such as Internet Explorer 7.0 or later, Google Chrome, Apple Safari work but some features may not display as intended. HIVToolbox is free for academic use, but a license is required for non-academic use.

In the future, we plan to integrate known drug binding sites and drug resistance mutations into the database and the structural viewers. We also plan to update the database with more minimotifs as they are annotated and the application will be adapted to upload user-generated multiple sequence alignments. A user registration feature will allow personalized displays and data storage. We also plan in include an epidemiology module.

## Methods

### New structural models for Integrase

To generate structural models we used superposition backbone atoms of existing HIV-1 IN structures using Molmol; superposition of all IN domains yielded RMSD of backbones residues less than = 0.4 Å [36]. To create a model of the 3-domain IN monomer, the catalytic domains of IN structures from 1EX4 (NTD and CCD) and 1K6Y (CCD and CTD) were superimposed (called INTM) [8], [10]. The CCD domains of two sets of monomers were fit to the dimer structure of 1EX4 (called INTD). To generate tetramers, the two IN dimers were fit to the CCD domains of the 1K6Y tetramer (RMSD = 0.4 Å; called INTT). Finally, the CCDs of INTT were fit to the CCDs of 4IN:LEDGF hetero-dimers (2B4J) to create an IN:LEDGF hetero-octamer (called IN4L) [26]. In these models the domains of IN are tightly packed and there is no observable van der Waals overlap between atoms.

The NTD, CCD and CTD domains of HIV-1 and PFV IN have highly similar folds, however the peptide linkers between these domains are of different lengths and have different structures [PDB: 1EX4, 1K6Y, 1WJA] [8], [10], [11], [36]. We spatially fit the HIV-1 IN domains to the PFV tetramer, without the interdomain linkers. Since the PFV structure (3LQ2) is missing the NTD and CTD in the second subunit of the dimer, we modeled these based on the structure of the complete HPV monomer. This produced a structural tetramer model of full length HIV-1 IN bound to two ends of viral DNA (IN42). Superposition of the IN catalytic domains in the IN:LEDGF structure complex (2B4J) to these domains in IN42 yielded the IN:LEDGF hetero-octamer bound to two viral DNAs (INL2).
